# Sources, morphology, phytochemistry, pharmacology of *Curcumae Longae Rhizoma*, *Curcumae Radix*, and *Curcumae Rhizoma*: a review of the literature

**DOI:** 10.3389/fphar.2023.1229963

**Published:** 2023-08-31

**Authors:** Xin Zhu, Yun-yun Quan, Zhu-jun Yin, Min Li, Ting Wang, Lu-yao Zheng, Shi-qi Feng, Jun-ning Zhao, Li Li

**Affiliations:** ^1^ Sichuan Academy of Chinese Medicine Sciences, Sichuan Institute for Translational Chinese Medicine, Biological Assay Key Laboratory of State Administration of Traditional Chinese Medicine for Traditional Chinese Medicine Quality, Translational Chinese Medicine Key Laboratory of Sichuan Province, Sichuan Engineering Technology Research Center of Genuine Regional Drug, Engineering Research Center for Formation Principle and Quality Evaluation of Genuine Medicinal Materials in Sichuan Province, Chengdu, China; ^2^ Chengdu University of Traditional Chinese Medicine, School of Pharmacy, Chengdu, China

**Keywords:** *Curcumae Longae Rhizoma*, *Curcumae Radix*, *Curcumae Rhizoma*, pharmacology, curcumin, targets

## Abstract

*Curcumae Longae Rhizoma* (turmeric), *Curcumae Radix* and *Curcumae Rhizoma* are derived from the *Curcuma* species, and have gradually become three of the most commonly used medicinal herbs in China due to their different origins, processing methods and medicinal part. These three herbs have certain similarities in morphology, chemical composition, and pharmacological effects. All three of these herbs contain curcuminoids and volatile oil compounds, which exhibit anti-inflammatory, anti-tumor, antioxidant, and neuroprotective properties, although modern clinical applications have their own requirements. At present, there is no systematic guidelines for the clinical application of these three of *Curcuma* species; consequently, there is a high risk of unwanted phenomena associated with the mixing and indiscriminate use of these herbs. In this review, we focus predominantly on morphology, chemical composition, and the pharmacological activity of these three *Curcuma* herbs and summarize the current status of research in this field. Our goal is to provide a better understanding of clinical value of these *Curcuma* species so that we can provide reference guidelines for their further development, utilization and rational clinical application.

## 1 Introduction

**FIGURE 1:**
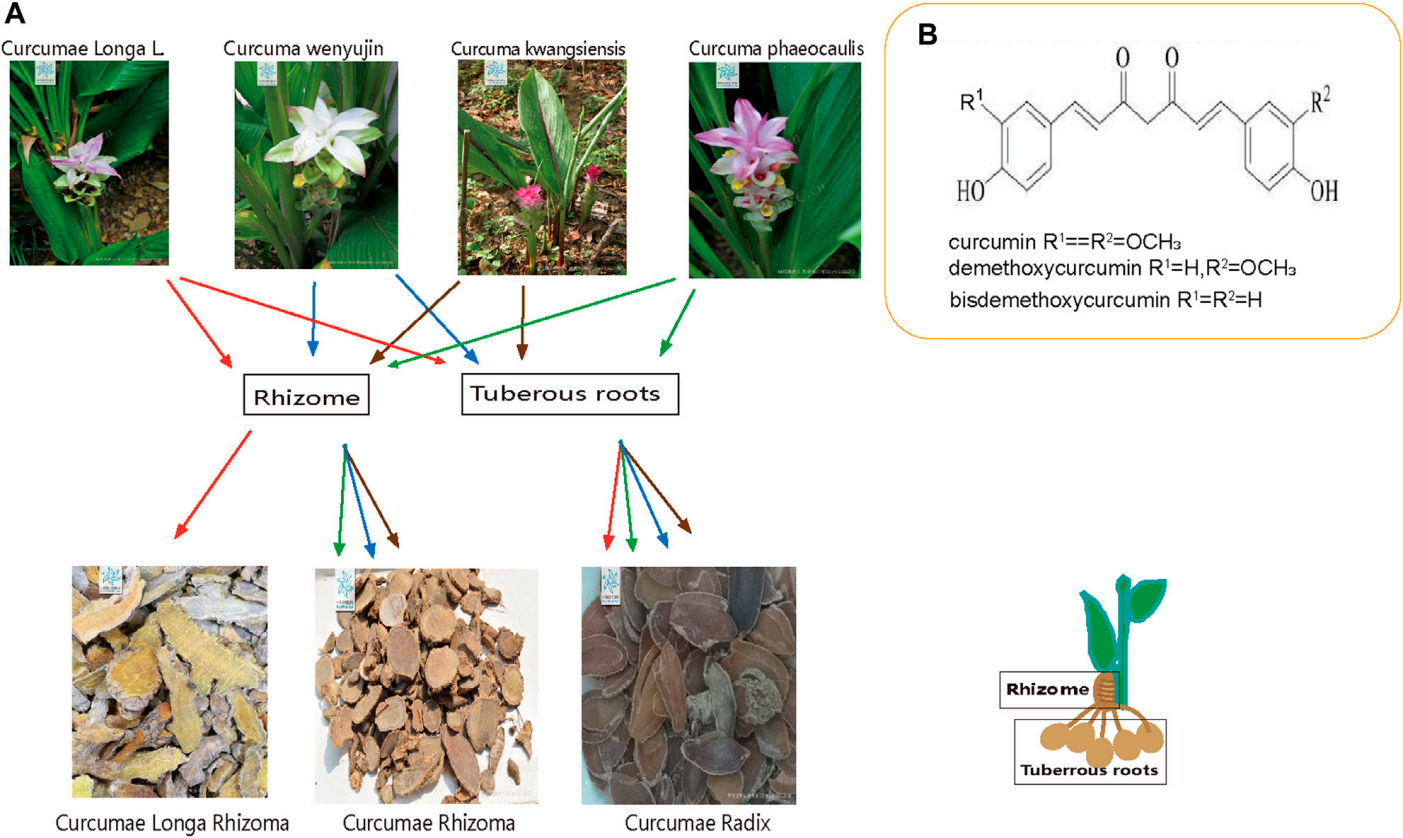
Overview of the three plants. **(A)** Relationship between *Curcumae Longae Rhizoma, Curcumae Radix and Curcumae Rhizoma* **(B)** chemical structure formula of Curcumin, demethoxycurcumin and bisdemethoxycurcumin.

The commonly used traditional Chinese medicines *Curcumae Longae Rhizoma*, *Curcumae Radix* and *Curcumae Rhizoma* are derived from medicinal plants of the *Curcuma* species in the Zingiberaceae family and have been the focus of much research due to their commercial and medicinal values. *Curcumae Longae Rhizoma* has been used in Asia for thousands of years; indeed, the *Curcuma* species has been used in traditional Chinese medicine for more than a thousand years. These three forms of medicinal herbs were clearly distinguished during the Ming and Qing dynasties, and continue to be widely applied due to their medicinal properties (Chen, 1981). The 2020 edition of the Chinese Pharmacopoeia reported that *Curcumae Longae Rhizoma* is the dried rhizomes of *Curcuma Longa* L. of the Zingiberaceae family, and that Sichuan is the main Taoist producing area for *Curcumae Longae Rhizoma* in China. *Curcumae Radix* is the dried tuberous roots of *Curcuma wenyujin* (Y. H. Chen and C. Ling), *Curcuma Longa* L., *Curcuma kwangsiensis* (S. G. Lee and C. F. Liang), and *Curcuma phaeocaulis* Val. of the ginger plant family; these herbs are habitually known as “Wenyu-jin,” “Huangyujin,” “Guiyujin” and “lvyu-jin,” and are mainly produced in Jiangsu, Zhejiang, Guangxi, Sichuan, Yunnan and other places in China. *Curcumae Rhizoma* is the dried rhizome of *C. phaeocaulis* VaL., *C. kwangsiensis* (S. G. Lee and C. F. Liang), *C. wenyujin* (Y. H. Chen and C. Ling) of the family of Zingiberaceae (Chinese Pharmacopoeia Commission, 2020), and is mainly produced in Fujian, Guangdong, Zhejiang, Sichuan, and Yunnan in China. *Curcuma phaeocaulis*, *C. kwangsiensis*, and *C. wenyujin* can be used for multiple purposes at the same time; a common phenomenon in traditional Chinese Medicine. The tuberous roots of these four *Curcuma* species can be used as *Curcumae Radix*, while the rhizomes of *C. phaeocaulis*, *C. kwangsiensis*, and *C. wenyujin* can be used as *Curcumae Rhizoma*; the rhizomes of *Curcuma Longa* L. are used as *Curcumae Longae Rhizoma*. Consequently, it is highly evident that *Curcumae Longae Rhizoma*, *Curcumae Radix* and *Curcumae Rhizoma* are inextricably linked to each other, and demonstrate both correlations and differences in terms of their properties and abilities; the relationships that exist between these three herbs is shown in Figure 1A.

The curcuminoids and volatile oil compounds possessed by *Curcuma* species have been widely studied due to their significant biological activities, their numerous pharmacological effects, including (anti-inflammatory, antitumor, antioxidant, and antimicrobial), their ability to reduce blood glucose levels, and their therapeutic effects on the nervous, digestive and cardiovascular systems (Anand et al., 2008; Kunnumakkara et al., 2008; Rathore et al., 2008; Lekshmi et al., 2012; Gadnayak et al., 2022; Alameri et al., 2023; Balakumar et al., 2023; Erarslan et al., 2023; Jabbar et al., 2023; Louisa et al., 2023; Mohanty et al., 2023; Wei et al., 2023). Differences in the chemical composition and pharmacological effects of *Curcuma* species are caused by variations in growth environment, medicinal components, cultivation methods and processing, and a range of other factors. These differences allow us to utilize the resources of these three medicinal herbs more effectively and eliminate the phenomena associated with the mixing and indiscriminate use of such herbs. In this review, we summarize the literature relating to plant sources, chemical composition, pharmacological effects and other aspects of *Curcumae Longae Rhizoma*, *Curcumae Radix* and *Curcumae Rhizoma*. In addition, we analyze the similarities and differences between the three herbs and attempt to provide specific guidelines for the future investigation and utilization of *Curcuma* species and to provide a basis for the rational use of these herbs in clinical scenarios.

## 2 Manuscript formatting

### 2.1 Character identification and microscopic identification

*Curcumae Longae Rhizoma*, *Curcumae Radix* and *Curcumae Rhizoma* have their own features in character identification and microscopic identification. The Chinese Pharmacopoeia describes the character identification and microscopic identification features of *Curcumae Longae Rhizoma*, *Curcumae Radix* and *Curcumae Rhizoma* (Chinese Pharmacopoeia Commission, 2020). The differences in the character identification and microscopic identification features between the three of herbs are shown in Table 1.

**TABLE 1:** The character identification and microscopic identification of *Curcumae Longae Rhizoma, Curcumae Rhizoma* and *Curcumae Radix*.

Chinese herbal medicine		Character identification	Microscopic identification
*Curcumae Longae Rhizoma*	*Curcuma Longa* L.	(1) shape: irregularly ovoid, cylindrical or fusiform, often curved, some with short forked branches	(1) epidermal cells: flattened, thin-walled
		(2) length: 2–5 cm, diameter:1–3 cm	(2) cortex: broad, with leaf-traced vascular bundles
		(3) surface of herbs: dark yellow, rough, with crinkled texture and distinct links, with rounded branching scars and whisker scars	(3) cork cells: 6 to 8 rows on outer side near epidermis, flattened
		(4) cross-section of herbs: brownish-yellow to golden-yellow, keratinous, with waxy luster, the rings of endodermis evident, vascular bundles scattered in dots	(4) thin-walled cells contain oil droplets, starch granules and reddish-brown pigments
*Curcumae Rhizoma*	*Curcuma phaeocaulis*	(1) shape: oval, oblong-ovate, conical or long fusiform, more bluntly pointed at the tip, bluntly rounded at the base	(1) more cork cells
		(2) length: 2–8 cm, diameter:1.5–4 cm	(2) less leaf traces vascular bundles
		(3) surface of herbs: gray-yellow to gray-brown, upper links raised, with rounded, slightly concave fibrous root scars or residual fibrous roots	(3) endodermis evident
		(4) cross-section of herbs: grayish brown to bluish brown, waxy, often with grayish brown powder, cortex and mesostyle readily separable, endothelial annulus tan	(4) thin-walled cells filled with pasty starch granule clusters
	*Curcuma kwangsiensis*	(1) surface of herbs: slightly raised rings	(5) thin-walled tissue with cells containing golden-yellow oil scattered
		(2) cross-section of herbs: yellowish brown to brown, often with yellowish powder, inner cortical rings yellowish white	(6) vessel: mostly threaded and trapezoidal, 20–65 μ m in diameter.
	*Curcuma wenyujin*	(1) cross-section of herbs:Yellowish brown to tan, often with light yellow to yellowish brown powder	(7) fiber pore grooves are obvious, 15–35 μ m in diameter
*Curcumae Radix*	*Curcuma wenyujin*	(1) shape: oblong or ovoid, slightly compressed, some slightly curved, both ends acuminate	(1) velamen:Narrow, in 4–8 rows of cells, thin-walled, slightly undulating, neatly arranged
		(2) length:3.5–7 cm, diameter:1.2–2.5 cm	(2) less oil cells
		(3) surface of herbs: grayish brown, with irregular longitudinal wrinkles	(3) obvious endodermis
		(4) cross-section of herbs:Grayish brown, keratinous, endodermal ring conspicuous	(4) vessel: 2 to 4 xylem bundle ducts, ducts are polygonal, thin-walled, 20–90 μ m in diameter, and have microlignified fibers
			(5) pasteurized starch granules visible in thin-walled cells
	*Curcuma kwangsiensis*	(1) shape: long conical or oblong in shape	(1) velamen: occasionally thickened with 1 or 2 rows of thick-walled cells
		(2) length: 2–6.5 cm, diameter:1–1.8 cm	(2) vessel: circular, up to 160 μ m in diameter
		(3) surface of herbs: with sparse shallow longitudinal lines or rougher reticulate wrinkles	
	*Curcuma phaeocaulis*	(1) shape: long oval in shape, thicker	(1) velamen:thickening-free
		(2) length: 1.5–3.5 cm, diameter:1–l.2 cm	(2) oblate vessel
			(3) pigmented cells are often found at the outer cortex of the middle column
	*Curcuma Longa* L.	(1) shape: fusiform, some with elongated ends	(1) velamen:thickening of the innermost cell wall
		(2) length: 2.5–4.5 cm, diameter:1–1.5 cm	(2) more oil cells
		(3) surface of herbs: brownish gray or grayish yellow, finely wrinkled	(3) pigment cells scattered everywhere in the thin-walled tissue
		(4) cross-section of herbs: orange-yellow, peripheral tan to brownish-red	

### 2.2 Chemical composition of *Curcumae Longae Rhizoma, Curcumae Radix* and *Curcumae Rhizoma*

There are several chemical constituents that are common to *Curcumae Longae Rhizoma Curcumae Radix* and *Curcumae Rhizoma*; these predominantly include curcuminoids, volatile oils, sugars, and sterols. *Curcumae Longae Rhizoma* and *Curcumae Radix* also contain flavonoids, organic acids, alkaloids, polypeptides, and a variety of trace elements, including Ca, K, Na, Zn, Cu, Mn, Pb, and Cd (Zeiner et al., 2022; Yin et al., 2012). Curcuminoids and volatile oils are the major active constituents of *Curcuma* species and account for the largest proportion of all components. Curcuminoids is a collective term used to describe the diarylheptanoids that feature in the chemical structural formula. This term is also used to refer to the structural similarity of the diarylheptanoids; curcumin, demethoxycurcumin and bisdemethoxycurcumin account for the three largest proportions of active ingredients in this class of compounds. Chemical formulas for the three chemical compounds are shown in Figure 1B. The content of curcuminoids in *Curcumae Longae Rhizoma* is higher than that in the other two herbs. A previous study found that the content of curcumin was highest in *Curcumae Longae Rhizoma*; this was followed by *Curcumae Rhizoma*; the lowest content was in *Curcumae Radix* (Sun et al., 2017). More than 40 curcuminoids have been isolated and identified (Claeson et al., 1993; Jurgens et al., 1994; Lin and Lin-Shiau, 2001; Zeng et al., 2007; Jiang et al., 2009; Xing et al., 2009); of these, 30 have been detected in *Curcumae Longae Rhizoma* (Zhou, 2015), 20 in *Curcumae Radix* (Hu and Shao, 2007), and 36 curcuminoids in *Curcumae Rhizoma*. More than 39 types of volatile oil compounds have been identified in *Curcumae Longae Rhizoma* (Ge et al., 2007; Li et al., 2009; Wu et al., 2011; Cui et al., 2016; Lu, 2018; Wei et al., 2020); these are mostly sesquiterpenes or monoterpenes, such as ar-turmerone, α-turmerone, α-curcumene, and β-sesquiphellandrene. The volatile oil compounds of *Curcumae Radix* contain 69 terpenes, mostly sesquiterpenes, monoterpenes, and diterpenes; of these, sesquiterpenes account for the largest proportion (Yin et al., 2012; Liu et al., 2021), including germacrone, furanodiene, β-pinene, isoborneol, and α-terpineol. *Curcumae Rhizoma* contains 63 volatile oil compounds, predominantly including curcumol, curcumone, furanodiene, curdione, and β-elemene (Huang et al., 2020; Li et al., 2021). The main chemical constituents of *Curcumae Longae Rhizoma, Curcumae Radix* and *Curcumae Rhizoma* are shown in Table 2.

**TABLE 2:** Study on chemical constituents of *Curcumae Longae Rhizoma, Curcumae Radix* and *Curcumae Rhizoma*.

Chinese herbal medicine	Chemical composition	Content detection
*Curcumae Longae Rhizoma*	curcumin (Liu et al., 2022),demethoxycurcumin (Liu et al., 2022), bisdemethoxycurcumin (Liu et al., 2022), ar-turmerone (Manzan et al., 2003), α-turmerone (Manzan et al., 2003), β-turmerone (Manzan et al., 2003), β-caryophyllene (Manzan et al., 2003), eucalyptol (Manzan et al., 2003), α-phellandrene (Manzan et al., 2003), (−)-zingiberene (Tang et al., 2022), β-sesquiphellandrene (Tang et al., 2022), α-curcumene (Tang et al., 2022), terpinolene (Tang et al., 2022), (−)-β-Pinene (Tang et al., 2022), α-Terpinene (Tang et al., 2022), D-Limonene (Tang et al., 2022), Eucalyptol (Tang et al., 2022), Terpinolene (Tang et al., 2022), Linalool (Tang et al., 2022), α-Terpineol (Tang et al., 2022), (E)-β-Farnesene (Tang et al., 2022), γ-Curcumene (Tang et al., 2022)	curcumin1.0%
		volatile oil7.0% (ml/g) (Chinese Pharmacopoeia Commission, 2020)
*Curcumae Radix*	curcumin (Chen et al., 2017), demethoxycurcumin (Chen et al., 2017), bisdemethoxycurcumin (Chen et al., 2017), (−)-zingiberene (Tang et al., 2022), β-sesquiphellandrene (Tang et al., 2022), terpinolene (Tang et al., 2022), (−)-β-Pinene (Tang et al., 2022), Camphene (Tang et al., 2022), α-Terpinene (Tang et al., 2022), D-Limonene (Tang et al., 2022), Eucalyptol (Tang et al., 2022), Terpinolene (Tang et al., 2022), α-Terpineol (Tang et al., 2022), (E)-β-Farnesene (Tang et al., 2022), γ-Curcumene (Tang et al., 2022), Camphor (Liu, 2006), Borneol (Liu, 2006), Isoborneol (Liu, 2006), α-Bulnesene (Liu, 2006), β-Elernenone (Liu, 2006), Aristolene (Liu, 2006), β-Elemerle (Liu, 2006), Curcumol (Liu, 2006), 1-Pentadecanol (Liu, 2006)	-
*Curcumae Rhizoma*	curcumin (Zang et al., 2021), demethoxycurcumin (Zang et al., 2021), bisdemethoxycurcumin (Zang et al., 2021), curdione (Ren and Li, 2013), curcumenol (Ren and Li, 2013), germacrone (Ren and Li, 2013), curzerene (Ren and Li, 2013), furanodiene (Ren and Li, 2013), β-elemene (Ren and Li, 2013), isocurcumenol (Yu, 2021), furanodienone (Yu, 2021), α-pinene (Wang et al., 2021), curzerene (Wang et al., 2021), camphene (Wang et al., 2021), caryophyllene (Wang et al., 2021), curcumenone (Wang et al., 2021), D-Limonene (Wang et al., 2021), Eucalyptol (Wang et al., 2021), Linalool (Wang et al., 2021)	volatile oil1.0% (ml/g) (Chinese Pharmacopoeia Commission, 2020)

### 2.3 The pharmacological effects of *Curcumae Longae Rhizoma*, *Curcumae Radix*, and *Curcumae Rhizoma*

#### 2.3.1 Anti-inflammatory and analgesic effects

Inflammation is a defense response of the body to stimuli and is manifested by redness, swelling, heat, pain and dysfunction. In the normal human body, the levels of cytokines and chemokines are always low; it is only when the body repairs the physical damage caused by injury or infection that the levels of cytokines in the body, such as tumor necrosis factor-α (TNF-α), interleukin-6 (IL-6), and nitric oxide (NO), increase, ultimately leading to the development of inflammation (Dell'Agli et al., 2013). Curcuminoids and volatile oil compounds exert significant anti-inflammatory effects that are comparable to those of steroidal or non-steroidal anti-inflammatory drugs (Guo et al., 2022). In a previous study, Wang et al. (Wang Z. et al., 2022) explored the effect of curcumin on *Mycoplasma* pneumoniae (MP)-infected pneumonic BALB/c mice based on the NF-κB pathway; analysis showed that the expression of NF-κBp65 protein was reduced in the lung tissues of the mice in the medium-dose and high-dose groups of curcumin, and that the levels of IL-6, IL-8 and TNF-α were significantly reduced in the bronchial lavage fluid (*p* < 0.01), thus alleviating the symptoms of pneumonic mice. In another study, Funk et al. (Funk et al., 2010) explored the therapeutic effect of turmeric essential oil on streptococcal cell (SCW)-induced arthritis in female Lewis rats, and ultimately found that turmeric volatile oil significantly alleviated SCW-induced arthritis in female Lewis rats; the mechanism of action was considered to involve blockade of the intra-articular inflammatory cells (neutrophil chemokine GRO/KC and monocyte chemokine MCP-1) and the expression levels of the key inflammatory cytokine interleukin-1β (IL-1β) as well as other downstream joint inflammatory mediators (e.g., COX-2). In other research, Li et al. (Liju et al., 2011) investigated the anti-inflammatory and analgesic effects of turmeric volatile oil and used gas chromatography-mass spectrometry (GC-MS) to analyze the material basis of the anti-inflammatory and analgesic effects generated by the volatile oil of turmeric, which showed better anti-inflammatory and analgesic effects when compared with aspirin and diclofenac. The main components of turmeric volatile oil were ar-turmerone, curlone and ar-curcumene. In a previous study, Henrotin et al. (Henrotin et al., 2019) compared the therapeutic effects of different doses of *Curcumae Longae Rhizoma* extract on knee osteoarthritis (OA) and found that both low and high doses of *Curcumae Longae Rhizoma* extract led to a significant improvement in the patients’ general assessment of disease activity (PGADA), knee injury and osteoarthritis outcome score (KOOS), and reduced the serum levels of collagen 2-1 (Coll2-1) in patients with OA. Mukophadhyay et al. (Mukhopadhyay et al., 1982) demonstrated the anti-inflammatory activity of curcumin and other semi-synthetic analogs (sodium curcuminate NaC, diacetyl curcumin DAC, triethyl curcumin TEC, and tetrahydro curcumin THC) by investigating carrageenan-induced paw edema and cotton pellet granuloma in experimental rats.

Chen et al. (Chen et al., 2019) used GC-MS to analyze the chemical composition of the active components of *Curcumae Radix* extracts that relate to blood activation and analgesic efficacy; ultimately, curcumin and its derivatives were found to be the main bioactive components responsible for these effects. Qin et al. (Qin et al., 2022a) revealed that *Curcumae Radix* extract could treat dysmenorrhea by regulating the inflammatory reaction, relaxing smooth muscle and endocrine by curcumenone, 13-hydroxygermacrone (+)-cuparene, caryophyllene oxide, zederone, and isocurcumenol. In another study, Qiu (Qiu et al., 2014) found that the ethyl acetate extract of Wenyu-jin reduced the levels of TNF-α, IL-6, and IL-1β in ear tissues, and inhibited acute inflammation and the pain triggered by physical and chemical factors, inflammation-causing agent-induced ear swelling, and changes in capillary permeability; the inhibitory effect of this extract was stronger when the dosages was increased. Dong et al. (Dong et al., 2015) isolated a sesquiterpenoid compound (curcumolide) from Wenyu-jin with a unique structure that exhibited significant anti-inflammatory effects by inhibiting lipopolysaccharide (LPS)-induced NF-κB activation in RAW264.7 macrophages, and by reducing the production of TNF-α, IL-6, IL-1β, NO, and reactive oxygen species (ROS). In addition, Huang et al. (Huang et al., 2013) found that diterpenoid compound C from Wenyu-jin inhibited the secretion of pro-inflammatory factor IL-8, the expression of Ik Kα and Ik Kβ, and promoted the secretion of anti-inflammatory factor IL-4 in GES-1 gastric cells; these effects were enhanced when drug dosage was increased. Furthermore, this compound exerted an inhibitory effect on the inflammation induced by *Helicobacter pylori* (Hp) in human gastric GES-1 epithelial cells. Zheng et al. (Zhen et al., 2022) used Western blotting, immunofluorescence, enzyme linked immunosorbent assay (ELISA) and reverse transcription-PCR (RT-PCR) to investigate the effect of curcumol on macrophage M1/M2 phenotypic differentiation and its mechanism of action; analysis showed that curcumol could inhibit the inflammatory factors IL-1β and TNF-α, the M1 macrophage markers iNOS, NF-κB, and NLRP3 in activated RAW264.7 macrophages, and increased the expression of the M2 type macrophage marker CD206; these events, inhibited the inflammatory response by regulating macrophage phenotypic shift. Another investigation showed studied that a high dose extract group of *Curcumae Rhizoma*, and the high and low dose groups of *Curcumae Rhizoma* vinegar, significantly increased the levels of PGE2, 6-keto-PGF-1α, NO, and β-EP in uterine tissues, and reduced the levels of TXB2, PGF2α, IL-6, TNF-α, and Ca^2+^; these events exerted analgesic effects by regulating the levels of these pain factors (Tong, 2021). Information relating to the parameters described in pharmacological studies on the anti-inflammatory and analgesic effects of *Curcumae Longae Rhizoma*, *Curcumae Radix* and *Curcumae Rhizoma* is shown in Table 3.

**TABLE 3:** Pharmacological parameters of anti-inflammatory and analgesic effects of *Curcumae Longae Rhizoma, Curcumae Radix* and *Curcumae Rhizoma*.

Active extract	Animal models and inducers	Experimental type	Experimental grouping and administration method	Result	References
curcumin	Pneumonia mice (MP)	*In vivo*	curcumin (50, 100, 200 mg/kg), (ig)	NF-κBp65↓, IL-6↓, IL-8↓, TNF-α↓	Wang et al. (2022a)
turmeric volatile oil	Arthritis rats (SCW)	*In vivo*	turmeric volatile oil (56 mg/kg/d), (ip)	Joint swelling rate↓	Funk et al. (2010)
turmeric volatile oil	analgesic model mice (0.5% ethylic acid) and Inflammatory mice (2% formalin))	*In vivo* and *In vitro*	blank control group, aspirin100 mg/kg or diclofenac10 mg/kg, turmeric volatile oil (100, 500, 1,000 mg/kg), (ip and po)	inhibition rate of foot swelling↑, Pain threshold value↑, twisting frequency↓	Liju et al. (2011)
*Curcumae Longae Rhizoma* extract	OA patient	*In vivo*	Placebo group (2 ×3 piece/d ), BCL low (2 ×2 piece/d+2×1 piece/d Placebo ), high (2 ×3 piece/d ) dose intervention group, (po), (each capsule contains 46.67 mg *Curcumae Longae Rhizoma* extract)	Improving overall patient disease activity assessment (PGADA) and knee joint injury and osteoarthritis outcome score (KOOS), collagen 2-1 (Coll2-1) levels↓	Henrotin et al. (2019)
curcumin and other semi-synthetic analogs	Rat paw edema and inflammation cotton ball granuloma model (carrageenan)	*In vivo*	blank control group, C, NaC, DAC, TEC, THC(3, 10, 30, 60, 80 mg/kg)group, PB, FA (10, 30, 60 mg/kg) group	Foot swelling rate↓, granulation tissue weight↓	Mukhopadhyay et al. (1982)
*Curcumae Radix* extract	SPF grade analgesic model mice (0.8% ethylic acid)	*In vivo*	Different solvent extract groups (volatile oil 9.75 μ L/kg, petroleum ether 0.949 g/kg, ethyl acetate 1.43 g/kg, 1-butanol 0.403 g/kg, water 17.134 g/kg), blank control group (2%Tween-80), aspirin suspension 200 mg/kg), (ip)	Pain threshold value↑, torsion latency↑, torsion time↓, whole blood shear viscosity↓, red blood cell aggregation index↓, carson viscosity in PeG, EaG, BuG↓	Chen et al. (2019)
Guiyujin extract	Analgesic model Kunming mice (0.6% acetic acid) and ear swelling model (xylene)	*In vivo*	Guiyujin water extract and alcohol extract group (8, 16 g/kg), 0.2% Luotongding, blank control group, (ip)	Pain threshold value↑, twisting frequency↓, the swelling of the auricle↓, capillary permeability↓, mouse cotton ball granuloma hyperplasia↓	Qin et al. (2022a)
Wenyu-jin ethyl acetate extract	Analgesic model ICR mice (glacial acetic acid) and ear swelling model (xylene)	*In vivo*	Wenyu-jin ethyl acetate extract group (100 mg/kg, 200 mg/kg, 400 mg/kg), blank control group, aspirin 45 mg/kg, (ip)	capillary permeability↓, TNF-α↓, twisting frequency↓, pain threshold time↑	Qiu et al. (2014)
*Cur*cumolide	RAW264.7 macrophage (LPS)	*In vitro*	Curcumolide group (2, 10, 20 μ M), hydrocortisone (10 μ M), blank control group、model group	TNF-α↓, IL-6↓, IL-1β↓, NO↓, ROS↓	Dong et al. (2015)
diterpenoid compound C	Human gastric epithelial GES-1 cells (Hp)	*In vitro*	diterpenoid compound C group (0, 5, 10, 20, 40, 80 mg/L), negative control group, model group, amoxicillin	The proliferation of human gastric epithelial cells GES-1↓, IL-8↓, IL-4↑, NF-κBp65↓, Ik Kα↓, Ik Kβ↓	Huang et al. (2013)
curcumenol	Mouse macrophage cell line RAW264.7 (LPS)	*In vitro*	blank control group, model group , curcumenol (12.5 mg/L, 25 mg/L, 50 mg/L)	IL-1β↓, TNF-α↓, IL-10↑, iNOS↓, CD206↑, NF-κB↓, NLRP3↓	Zhen et al. (2022)
*Curcumae Rhizoma* extract	SD rat primary dysmenorrhea model with qi stagnation and blood stasis (estradiol benzoate and 0.1% adrenaline hydrochloride)	*In vivo*	blank control group, model group, ibuprofen group (0.06 g/kg), Tongjingbao group (2.10 g/kg), *Curcumae Rhizoma* extract (3.80 g/kg, 0.95 g/kg), (ig)	Twisting latency↑, twisting time↓, PGE2↑, 6-keto-PGF-1α↑, NO↑, β-EP↑, TXB2↓, PGF2α↓, IL-6↓, TNF-α↓, Ca^2+^↓	Tong (2021)

#### 2.3.2 Antioxidant effect

Oxidative stress refers to an imbalance between the production of oxidants and antioxidants; when ROS is produced by the body, it can directly oxidize a range of macromolecules, including membrane lipids, structural proteins, enzymes and nucleic acids, thus leading to impaired cell function and cell death (Zhang W. et al., 2023). Kim et al. (Kim et al., 2021) found that water extracts of turmeric helped to inhibit the reduction of grip strength and muscle mass caused by muscle atrophy while reducing the activity of antioxidant enzymes as well as the protein levels of the muscle atrophy-related genes *MuRF-1* and *Atrogin1* in mice. In addition, this water extract reduced the levels of malondialdehyde (MDA) in muscle tissues, effectively alleviating the symptoms of Dexamethasone-induced muscular atrophy. In another study, Li et al. (Liju et al., 2011) investigated the antioxidant activities of the components of volatile oil from turmeric and discovered that the volatile oil of turmeric could significantly increase the levels of antioxidant enzymes, superoxide dismutase (SOD), glutathione (GSH), and GSH-Px in the blood, as well as the levels of glutathione sulfotransferase and SOD in the liver of experiment mice (*p* < 0.01); in addition, these components exerted antioxidant effects. Oxidative stress is a key pathogenic factor in osteoporosis; Zhao et al. (Zhao et al., 2022) cultured osteoblasts *in vitro* to create a model of oxidative stress for osteoblasts and explored the effect of curcumin on osteoblasts under oxidative stress. Analysis showed that the curcumin-treated groups showed enhanced proliferation of osteoblasts under oxidative stress, and also showed an inhibition of oxidative stress, as manifested by an increase in T-AOC, SOD and CAT levels; in addition, MDA and p-p38 MAPK levels were significantly reduced (*p* < 0.01) while the levels of Wnt5a were upregulated. The mechanism of action of curcumin may be to inhibit the activation of p38MAPK and promote the expression of the Wnt signaling pathway; these events may play a protective role in osteoblasts. In addition, other studies have shown that curcumin is mainly involved in skeletal muscle reconstruction and actus through the oxidative pathways by inhibiting fibrosis and the apoptosis of skeletal muscle cells (Deng, 2022; Hu, 2022).

Li et al. (Li Y. et al., 2022) evaluated the antioxidant activity of eight neo-sesquiterpenoids isolated from Wenyu-jin with regards to activation of the Nrf2-ARE pathway in human embryonic kidney (HEK293) cells. Analysis showed that procurcumenol and 9-oxo-neoprocurcumenol possessed certain antioxidant activities which were mainly exerted through the activation of the Nrf2-ARE pathway in a dose-dependent manner. In addition, both Wenyu-jin extract and vitamin E have been shown to completely inhibit radiation-induced lipid peroxidation, and when compared with the traditional antioxidant vitamin E, Wenyu-jin extract had a stronger antioxidant effect (Wang et al., 1996). He et al. (He et al., 2013) established a model of H_2_O_2_-induced oxidative stress injury in human umbilical vein endothelial cell (HUVEC) cells and demonstrated the antioxidant effect of *Curcumae Radix* extract; analysis showed that *Curcumae Radix* extract exerted antioxidative stress activity and protective effected against endothelial injury. Gou et al. (Gou et al., 2015) investigated the scavenging ability and reducing ability of *C. phaeocaulis* polysaccharides by performing *in vitro* antioxidant experiments. Analysis showed that *C. phaeocaulis* polysaccharides had a significant scavenging effect and strong reducing ability on DPPH (1,1-Diphenyl-2-picrylhydrazyl radical) radicals, hydroxyl radicals and superoxide anions. Zhang et al. (Zhang et al., 2015) compared the antioxidant activities of *Curcumae Longae Rhizoma* and *Curcumae Rhizoma* by Folin-Ciocaheu and Aluminum Salt Chromatography and found that both herbs exhibited strong antioxidant capacity although the antioxidant capacity of *Curcumae Rhizoma* was slightly stronger than *Curcumae Longae Rhizoma*. Information on the parameters of pharmacological studies relating to the antioxidant effects of *Curcumae Longae Rhizoma*, *Curcumae Radix* and *Curcumae Rhizoma* are shown in Table 4.

**TABLE 4:** Pharmacological parameters of antioxidant effects of *Curcumae Longae Rhizoma*, *Curcumae Radix* and *Curcumae Rhizoma*.

Active extract	Animal models and inducers	Experimental type	Experimental grouping and administration method	Result	References
*Curcumae Longae Rhizoma* water extract	Muscle atrophy model ICR mice (DEX)	*In vivo*	blank control group, model group (DEX,1 mg/kg + exercise), *Curcumae Longae Rhizoma* water extract (1 g/kg/d + exercise), (ig)	grip strength ↓, muscle mass↓, MuRF-1↓, Atrogin1↓, MDA↓	Kim et al. (2021)
turmeric volatile oil	female Balb/C mice (4–6 weeks), weighing 20–25 g	*In vivo* and *In vitro*	blank control group, control group treated with paraffin oil only, turmeric volatile oil (100 mg/kg, 500 mg/kg), (po)	The IC_50_ of superoxide is 135 μ g/mL, and the IC_50_ of hydroxyl radical is 200 μ g/mL, SOD↑, GSH↑, GSH-Px↑	Liju et al. (2011)
curcumin	Osteoblast oxidative stress model (H_2_O_2_)	*In vitro*	blank control group, model group (50 μmol/L H_2_O_2_), curcumin (1.25, 2.5, 5, 10 μmol/L )	Osteoblast viability↑, T-AOC↑, SOD↑, CAT↑, MDA↓, Wnt5a↑, p-p38 MAPK↓	Zhao et al. (2022)
*Curcumae Radix* alcohol extract	Oxidative stress injury model of human umbilical vein endothelial cells (HUVEC) cells (H_2_O_2_)	*In vitro*	blank control group, model group (800 μM H_2_O_2_), *Curcumae Radix* alcohol extract (25 mg/L ,50mg/L, 100 mg/L), vitamin E group (0.1 mM)	SOD↑, LDH↑, MDA↓, ROS↓	He et al. (2013)
*Curcumae Rhizoma* polysaccharide	—	*In vitro*	different concentrations of *Curcumae Rhizoma* polysaccharide group, DPPH group, ascorbic acid group (VC)	The IC_50_ of DPPH free radical is 0.29 mg/mL, the IC_50_ of ·OH is 0.44 mg/mL, and the IC_50_ of superoxide anion is 0.36 mg/mL	Gou et al. (2015)

#### 2.3.3 Antitumor effect

Cancer is one of the leading causes of death globally, and lung, liver, stomach, breast, and colon cancers are the top five leading causes of cancer-related deaths (Zhang Y. et al., 2023). Liver cancer rose had the third-highest cancer mortality rate in 2018; this rose to the second-highest in 2020. It was estimated that China will account for 24% of the world’s newly diagnosed cases and 30% of cancer-related deaths in 2020 (Cao et al., 2021). Many studies have now shown that curcumin can be used as a safe and effective anticancer drug that exhibits inhibitory effects in a variety of cancers such as gastric (Zhang X. et al., 2020), breast (Ma et al., 2022), liver (Zuo et al., 2022), and prostate cancer (Cheng and Gao, 2022), and that its anti-tumor mechanism of action is through the effective induction of apoptosis, the prevention of metastasis and invasion, and by exerting influence on a variety of growth factor receptors and cell adhesion molecules (Wilken et al., 2011; Yallapu et al., 2014; Qiao et al., 2020). Lei (Lei, 2013) reported that turmeric volatile oil inhibited the proliferation of liver cancer cells (Bel-7402, HepG2, and SMMC-7721) mainly by activating the mitochondrial apoptotic pathway, down-regulating the expression of Bcl-2 in the Bel-7402 liver cancer cells, reducing the levels of cytochrome C in the mitochondria, and by up-regulating Bax and activating the expression of caspase-9- and caspase-3-induced apoptosis in liver cancer cells. Lee et al. (Lee et al., 2018) revealed that curcumin can effectively reduce the protein levels of SIRT1. With regards to mechanisms, the electrophilic α- and β-unsaturated carbonyl portion of curcumin has been shown to exert certain antioxidant activities. When combined with the SIRT1 protein, curcumin can promote the effects of SIRT1 on the proteasomal degradation of colorectal cancer and exert anti-tumor effects. In another study, Jiang et al. (Jiang et al., 2012) measured the anti-tumor inhibition activity of curcuminoids extracts on Hela cells of cervical cancer and identified the main active components that could effectively inhibit the tumor cells from 26 species of curcuminoids extracts. Analysis showed that 13 types of curcuminoids (including cyclo-curcumin, cyclo-demethoxycurcumin and cyclo-bisdemethoxycurcumin) could effectively inhibit the proliferation and metastasis of HeLa cells and concluded that the antitumor effect was more significant when the structural formula featured a conjugated system, carbonyl or hydroxyl group. Liu et al. (Liu et al., 2019) investigated the effects of Wenyu-jin extracts on A498 and Sw-156 cells (renal cell carcinoma) and analyzed its mechanism of action; analysis showed that Wenyu-jin extracts caused mitochondrial dysfunction in A498 renal cell carcinoma cells, thus promoting the release of cytochrome C enzymes and inducing apoptosis in A498 cells. β-Elemene is a natural compound; Deng et al. (Deng et al., 2019) investigated the inhibitory effects of β-elemene on tumor cells and found that β-elemene effectively inhibited the peritoneal spreading and metastatic ability of gastric carcinoma. It was considered that the mechanism of action of β-elemene involves the downregulation of FAK phosphorylation and by influencing the expression of Claudin-1.

**FIGURE 2:**
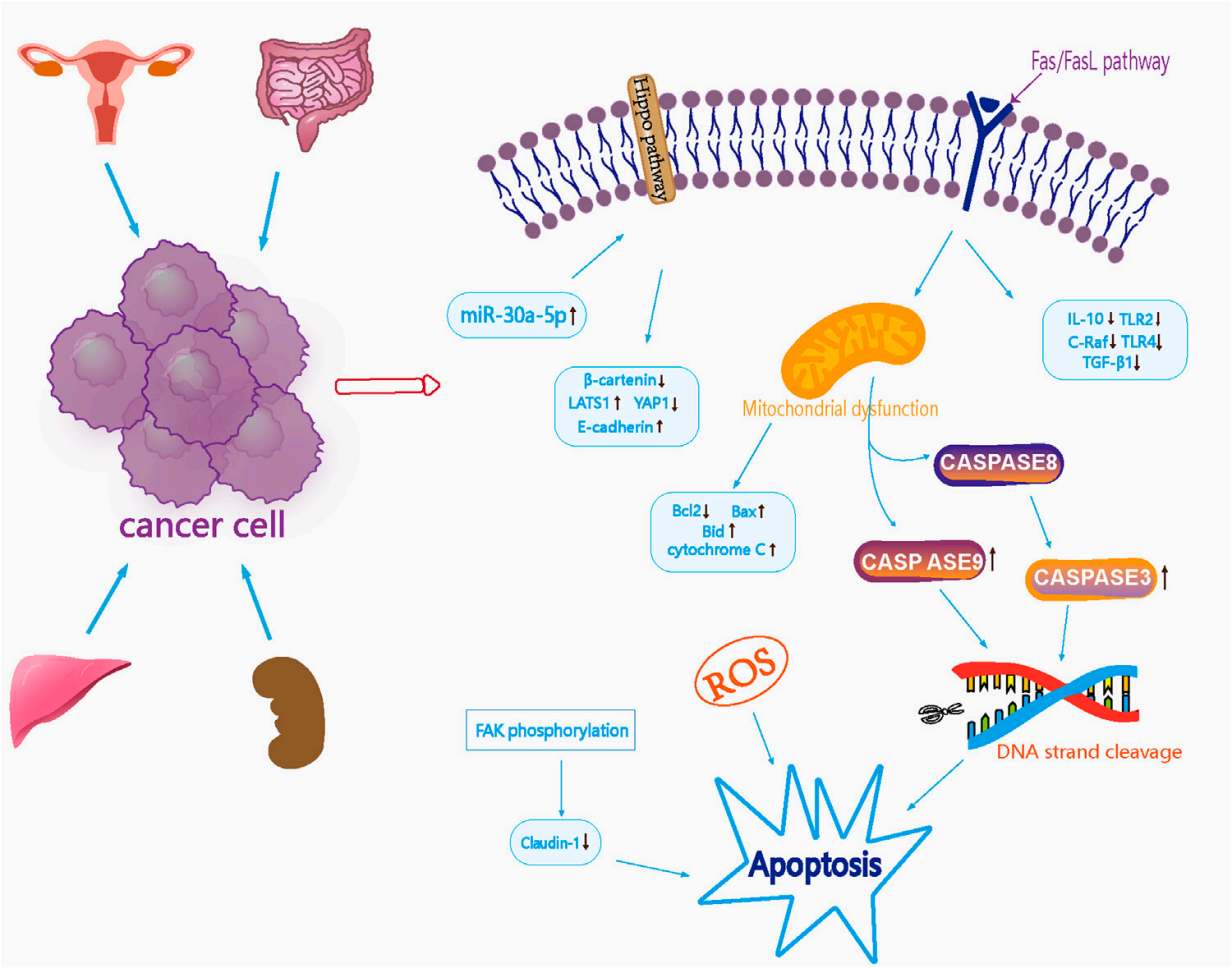
Mechanism diagram of antitumor effects of *Curcumae Longae Rhizoma, Curcumae Radix and Curcumae Rhizoma*.

Xu et al. (Xu et al., 2018) investigated the effects of *Curcumae Rhizoma* oil on the protein expression of death factor receptor (Fas), secreted immune factors (Toll-like receptor 2 (TLR2), Toll-like receptor 4 (TLR4), interleukin-10 (IL-10)), and the oncogene C-Raf transforming growth factor-beta 1 (TGF-β1) in SW1463 rectal cancer cells and found that the protein expression of IL-10, TLR2, TLR4, and C-Raf was significantly reduced in cells from the *Curcumae Rhizoma* oil concentration group (*p* < 0.01). In addition, the protein expression of TGF-β1 was significantly reduced (*p* < 0.05); an increasing dose of *Curcumae Rhizoma* oil exerted a stronger inhibitory effect on the proliferation of rectal cancer cells. It is possible that *Curcumae Rhizoma* oil may inhibit the proliferation of SW1463 rectal cancer cells by down-regulating the Fas/FasL pathway. The tumor suppressor microRNA-30a-5p is known to inhibit the proliferation of colon cancer cells; however, whether curcumol can inhibit colon cancer, and whether microRNA-30a-5p is involved, remains unknown. Yu et al. (Yu et al., 2021) investigated the effect of curcumol on colon cancer by MTT, Western blotting and PCR. With regards to the mechanism of action of curcumol on colon cancer, research showed that a reduction in the expression of the tumor suppressor microRNA-30a-5p, accompanied by inactivation of the Hippo signaling pathway, led to an improvement in the viability and migration rate of colon cancer cells. Furthermore, curcumol increased the expression of miR-30a-5p in cells and activated the Hippo signaling pathway, thus inhibiting the expression of YAP1, β-catenin and MMP2 in HCT116 cells. Furthermore, curcumol increased the expression of E-cadherin, MST-1, LATS1 and p-YAP1, and inhibited the proliferation and migration of colon cancer cells. Pharmacological parameters of the antitumor effects of *Curcumae Longae Rhizoma, Curcumae Radix and Curcumae Rhizoma* are shown in Table 5. In summary, it is highly evident that *Curcumae Longae Rhizoma, Curcumae Radix and Curcumae Rhizoma* can exert anticancer effects via complex signaling pathways, including the Hippo and Fas/FasL pathways, by the upregulation of LATS1, p-YAP1, Bid, and Bax levels, and by the downregulation of IL-10, TLR2, and TLR4 levels. These herbs can exhibit antitumor effects on several cancers, but particularly cervical, colon, renal and liver cancer; the anticancer mechanisms of these herbs are shown in Figure 2.

**TABLE 5:** Pharmacological parameters of antitumor effects of *Curcumae Longae Rhizoma*, *Curcumae Radix* and *Curcumae Rhizoma*.

Active extract	Tumor cell type	Experimental type	Experimental grouping	Result	References
Turmeric volatile oil	Human liver cancer cell lines (Bel-7402 cells, SMMC-7721 cells, and HepG-2 cells)	*In vitro*	①Blank control group、Turmeric volatile oil (10, 25, 50, 100, 200, 250 μg/mL )	cell viability↓, caspase-9↑, caspase-3↑, Bcl-2↓, Bax↑, Cytochrome C in mitochondria↑	Lei (2013)
			②Treatment of Bel-7402 cells with turmeric volatile oil (20, 40, 80 μg/m L)		
Curcumin	Colon cancer HCT-116 cells	*In vivo*	blank control group, Curcumin (50, 100 mg/kg), (ig)	SIRT1↓	Lee et al. (2018)
*Curcumae Radix* extract	Esophageal cancer TE-1 cells	*In vitro*	blank control group, *Curcumae Radix* extract (25 mg/L, 50 mg/L, 100 mg/L)	esophageal cancer TE-1 cell growth and proliferation↓	Jing et al. (2012)
*Curcumae Radix* extract	Renal cancer A498 cell line and Sw-156 cell line	*In vitro*	blank control group, *Curcumae Radix* extract (25 μg/mL, 50 μg/mL)	ROS↑, Cytochrome C ↑, caspase3↑, caspase9↑, Bid↑, Bax↑, Bcl2↓	Liu et al. (2019)
β-elemene	Tumor BGC823 and SGC7901 cell lines	*In vivo* and *In vitro*	blank control group, β-elemene group (1, 5 μ g/mL)	Claudin-1↓, FAK phosphorylation↓	Deng et al. (2019)
*Curcumae Rhizoma* Oil	Rectal cancer SW1463 cells	*In vitro*	blank control group、*Curcumae Rhizoma* Oil group (80, 120, 160, 200 mg/L)	IL-10↓, TLR2↓, TLR4↓, C-Raf↓, TGF-β1↓	Xu et al. (2018)
*Curcumae Rhizoma* Oil	Ovarian cancer patients	*In vivo*	combination group (400 mg *Curcumae Rhizoma* Oil + conventional chemotherapy), blank control group (conventional chemotherapy), (po)	The total effective rates were 76.67% and 56.67%, respectively. The incidence of adverse reactions ↓, improved the KPS score	Ruan and Zhong (2018)
curcumenol	cell lines of colon cancer HCT116 and SW620	*In vivo*	curcumenol (0, 10, 20, 40, 80, 100, 120, 160 μg/mL)	miR-30a-5p↑, YAP1↓, β-catenin↓, MMP2↓, E-cadherin↑, MST-1↑, LATS1↑, p-YAP1↑	Yu et al. (2021)

#### 2.3.4 Antibacterial effect

Microorganisms affect the human defense system in many different ways, with their impact ranging from mild to life-threatening conditions. These infections occur when harmful bacteria invade the body, leading to a host immune response that can result in localized or systemic symptoms (de Souza et al., 2023). Figueira et al. (W Figueira et al., 2020) investigated the effect of turmeric volatile oil on *Gram-positive bacteria*, *Gram-negative bacteria* and *fungi*. Analysis showed that the minimum inhibitory concentration (MIC) of *Staphyloccocus aureus Rosenbach* and *Pseudomonas aeruginosa* was 25 mg/mL while the MIC of *Candida albicans* was 12.5 mg/mL; thus, turmeric volatile oil was demonstrated to possess significant antimicrobial activity. In another study, Hu et al. (Hu et al., 2017) explored the antimicrobial effect and mechanisms of turmeric volatile oil and found that turmeric volatile oil had significant inhibitory effects on the growth of *Aspergillus flavus* with a certain dose-dependence. Paw et al. (Paw et al., 2020) concluded that the antimicrobial activity of turmeric volatile oil was superior to that of antimicrobial drugs such as fluconazole and ciprofloxacin, and had strong inhibitory effects on *Bacillus subtilis* (MIC: 7.5 μ g/mL), *Bacillus cereus* (MIC: 7.5 μ g/mL), and *fungi* (MIC: 2.5 μ g/mL); the strongest inhibitory effect was on *fungi*. Another study showed that the strength of the antimicrobial effect of chitosan/curnanocomplex (CS-Cur) was positively correlated with the content of curcumin (Mei et al., 2022). Zhu et al. (Zhu et al., 2013) used GC-MS to comparatively analyze the essential oils extracted from *Curcumae Longae Rhizoma*, *Curcumae Radix* and *Curcumae Rhizoma*, and tested the antimicrobial activities of the three essential oils and the six major constituents on two bacteria and one *fungi*. A total of 30 compounds were identified, including linalool, camphor, and β-elemene; the ratios of sesquiterpenes to monoterpenes in *Curcumae Longae Rhizoma*, *Curcumae Radix* and *Curcumae Rhizoma* were 2:1, 5:1, and 7:1, respectively. In addition, the MIC values of the essential oils and other constituents from the three herbs ranged from 62.5 to 500 μg /mL. The antibacterial activity of turmeric was stronger than that of the other two; this was attributed to the higher monoterpene content in turmeric. Zhang (Zhang T. et al., 2020) found that the alcoholic extract of *C. kwangsiensis* had significant antimicrobial activity against *Staphyloccocus aureus Rosenbach* and *P. aeruginosa*, with the inhibitory range can up to 10 mm against *Staphyloccocus aureus Rosenbach*, and with MIC/MBC values ranging from 391 to 6,250 μg/mL . Information relating to the parameters reported by pharmacological studies on the antimicrobial effects of *Curcumae Longae Rhizoma*, *Curcumae Radix* and *Curcumae Rhizoma* are shown in Table 6.

**TABLE 6:** Pharmacological parameters of antibacterial effects of *Curcumae Longae Rhizoma*, *Curcumae Radix* and *Curcumae Rhizoma*.

Active extract	Baterium	Experimental grouping, dosage and administration method	Result	References
*Curcumae Longae Rhizoma* extract	*Staphylococcus aureus*, *Pseudomonas aeruginosa*, *Candida* albicans	blank control group (serum-free medium), antibacterial agent group, *Curcumae Longae Rhizoma* extract group (*Staphylococcus aureus* 25 mg/mL, *Pseudomonas aeruginosa* 25 mg/mL, *Candida* albicans 12.5 mg/mL)	IL-1β↑, TNF-α↑, NO↑, The MIC of *Staphyloccocus aureus Rosenbach and Pseudomonas aeruginosa* is 25 mg/mL, while the MIC of *Candida albicans* is 12.5 mg/mL	W Figueira et al. (2020)
turmeric volatile oil	aflatoxin	turmeric volatile oil 1, 2, 3, 4, 8 μ L/mL	Spore germination rate↓, biosynthesis of ergosterol in *Aspergillus flavus*↓, mitochondrial ATPase activity↓, mitochondrial dehydrogenase↓	Hu et al. (2017)
turmeric volatile oil	*Bacillus subtilis*, *Bacillus* cereus, fungi	-	the MIC of *Bacillus subtilis* is 7.5 μ g/mL, the MIC of *Bacillus cereus* is 7.5 μ g/mL, the MIC of *fungi* is 2.5 μ g/mL	Paw et al. (2020)
chitosan/Curcumin nanocomplex	*Streptococcus* mutans	chitosan/curcumin nanocomplex (0, 0.01, 0.05, 0.10 mmol/L)	growth and proliferation ability of *Streptococcus*↓, glycolytic ability↓	Mei et al. (2022)
*Curcumae Rhizoma* ethanol extract	*Staphylococcus aureus*, *Candida* albicans, *Escherichia coli*, *Pseudomonas aeruginosa*	-	The antibacterial zone of *Staphyloccocus aureus Rosenbach* can reach 10mm, and its MIC/MBC value ranges from 391 ∼ 6250 μg/mL	Zhang et al. (2020b)

#### 2.3.5 Antiviral effect

Viral infections are prevalent in humans and can cause permanent injury to various degrees and by different mechanisms (Hammarström and Nyström, 2023). Liu et al. (Liu X. et al., 2023) investigated the effect of curcumin on porcine epidemic diarrhea virus (PEDV) infection by using VERO cells as experimental objects. Analysis showed that curcumin enhanced the antiviral effects of antiviral cytokines in VERO cells and inhibited the proliferation of PEDV in VERO cells by increasing the levels of IFN-β, MX1, ISG15, and ZAP. In addition, curcumin also inhibited a variety of intracellular signaling pathways, including mitogen-activated protein kinases (MAPKs), casein kinase II (CKII), and COP9 signalosome (CSN). Coxsackievirus (CVB3) infection has been associated with a variety of diseases, including myocarditis and dilated cardiomyopathy. The search for new antiviral drugs against CVB3 found that curcumin could significantly reduce viral RNA expression, protein synthesis, and viral titer, and to protect against virus-induced cytopathic effects and apoptosis. The mechanism by which curcumin acts might involve regulation of the dysregulated ubiquitin-proteasome system (UPS) to effectively inhibit the replication of CVB3 (Si et al., 2007). In another study, Dong et al. (Dong et al., 2013) conducted *in vitro* antiviral assays with sesquiterpenoids isolated from Wenyu-jin and found that Wenyu-jin extracts exerted significant antiviral activity against the influenza A virus and HIVI with IC_50_ values ranging from 6.80 to 39.97 μ M and SI values ranging from 6.35 to 37.25. Li (Li, 2018) investigated the effects and mechanisms of action of curcumol, curcumodione and germacrone on the H1N1 influenza virus and found that these medicines exerted protective effects on the early stages of H1N1-infected mice. These data corroborated the conclusion drawn from *in vitro* experiments in that the drugs mainly acted in the invasive and replicative phases of the virus, with germacrone having the most significant antiviral effect; furthermore, these antiviral effects were related to the MAPK signaling pathway. The pharmacological parameters of the antiviral effects of *Curcumae Longae Rhizoma*, *Curcumae Radix* and *Curcumae Rhizoma* are shown in Table 7.

**TABLE 7:** Pharmacological parameters of antiviral effects of *Curcumae Longae Rhizoma*, *Curcumae Radix* and *Curcumae Rhizoma*.

Active extract	Experimental subject	Virus type	Experimental grouping and dosage	Experimental method	Result	References
curcumin	VERO cell	porcine epidemic diarrhea virus (PEDV)	blank control group, model group, curcumin group, PEDV infection group after curcumin pretreatment	RT-qPCR, Western blot, IFA, Virus titer assay	Virus growth and proliferation↓, titer after virus infection↓, IFN-α↑, IFN-β↑、MX1↑, ISG15↑, ZAP↑	Liu et al. (2023a)
curcumin	HeLa cell	Coxsackie virus (CVB3)	blank control group, model group, curcumin (0, 10, 20, 30, 40 μM )	Western blot	Virus RNA↓, protein VP1↓, virus titer↓	Si et al. (2007)
curcumenol, curdion, germacrone	① Dog kidney cell line MDCK, African green monkey kidney cell VERO, human renal epithelial cell 293T, human non-small cell lung cancer A549 (*In vitro*), ② BALB/c female mouse (*In vivo*)	H1V1 virus	①curcumenol, curdion, germacrone group (1.56, 3.125, 6.25, 12.5, 25, 50, 100, 200 μmol/L), blank control group, solvent group, ②blank control group, model group, oseltamivir experimental group, curdion experimental group, and gemmazone experimental group	Quantitative PCR, Cellular Immunofluorescence, Western blot	H1V1 virus replication↓, liver and lung index↑, virus load↓, STAT1/2↓, IRF3/7↓, RIGI↓, PKR↓	Li (2018)

### 2.4 Effects of *Curcumae Longae Rhizoma, Curcumae Radix and Curcumae Rhizoma* on major systemic diseases

#### 2.4.1 Effects on the cardiovascular system

##### 2.4.1.1 Influence on hemorheology

According to the China Cardiovascular Health and Disease Report 2021, the number of individuals suffering from cardiovascular disease (CVD) in China is currently around 330 million. In addition, the prevalence of CVD is still increasing year-by-year, with CVD responsible for 46.74% and 44.26% of deaths in rural and urban areas in 2019 alone. This has led to new requirements for CVD prevention and treatment strategies and healthcare resource allocation in China (China Cardiovascular Health and Disease, 2022; Fu et al., 2023). Qiao et al. (Lei, 2013) explored the effects of turmeric extract on the cardiovascular system in New Zealand rabbits and diastolic isolated rat thoracic aorta, and concluded that turmeric extract could significantly inhibit platelet aggregation in rabbits (*p* < 0.05) and had a certain inhibitory effect on vascular ring contraction of the thoracic aorta in isolated rats caused by potassium chloride (KCl). Other research (Kim et al., 2012) demonstrated that curcumin and bisdemethoxycurcumin significantly prolonged the activated partialthromboplastin time (APTT) and prothrombin time (PT), and inhibited the activity of thrombin and activated factor X (FXa). The effect of curcumin was stronger than that of bisdemethoxycurcumin. Furthermore, it was deduced that methoxylation of the chemical structural formula enhanced its anticoagulant effect. Shah et al. (Shah et al., 1999) found that curcumin had a significant anticoagulant effect on blood vessels in the isolated rats, and inhibited the platelet aggregation induced by platelet-activating factor (PAF) and arachidonic acid (AA) in human blood, mainly by inhibiting the synthesis of thromboxane A2 (TXA2) and the Ca^2+^ signaling pathway. However, there was no significant inhibitory effect on the platelet aggregation induced by protein kinase C (PKC).

In other research, Jiang (Jiang et al., 2015) found that *Curcumae Radix* extract significantly inhibited platelet aggregation in rabbits and reduced the levels of TBIL, DBIL and AST in the serum of an experimental model of jaundice induced by α-naphthyl isothiocyanate (ANIT). Research has shown that the improvement of blood stagnation by Wenyu-jin extract is mainly related to lipid metabolism (linoleic acid metabolism, ether lipid metabolism, sphingolipid metabolism, glycerophospholipid metabolism, AA metabolism, and amino acid metabolism (including tryptophan metabolism and lysine degradation) (Hao et al., 2018). Su et al. (Su et al., 2019) found that the aqueous extract of Guiyujin (WECK) could maintain equilibrium of the ratio of TXA2 and PGI2 levels in the plasma, reduce the synthesis of TXA2 to inhibit platelet hyperfunction, promote the secretion of PGI2 by endothelial cells, prolong the activity of PT and APTT, induce vascular dilatation, and inhibit platelet aggregation (*p* < 0.01). Furthermore, the levels of TXB2 in the low-dose and medium-dose WECK groups were significantly elevated (*p* < 0.01); this was accompanied by a significant reduction in the levels of 6-K-PGF1α (*p* < 0.01). Furthermore, Guiyujin exerted antithrombotic effect by inhibiting the rate of thrombus formation by 38.23% and platelet aggregation by up to 72.10%. Chen et al. (Chen et al., 2018a; Chen et al., 2018b) demonstrated that the extract of *Curcumae Rhizoma* had a significant protective effect on death and hemiparesis caused by collagen-adrenaline-induced thrombosis in mice, significantly inhibited the formation of thrombi in the tail of mice caused by carrageenan, and reduced the number of animals that developed thrombosed black tails and the length of the black tails (*p* < 0.05, *p* < 0.01). These authors also demonstrated significant antithrombotic effects in venous blood vessels *in vivo*; the higher the concentration of *Curcumae Rhizoma* extract administered, the stronger the inhibitory effect. The antithrombotic effect of *Curcumae Rhizoma* extract may be related to increased levels of NO and 6-keto-PGF1α in the blood, reduced levels of ET-1 and TXB2, and reduced whole blood viscosity and plasma viscosity. Information on the parameters reported by pharmacological studies on the effect of *Curcumae Longae Rhizoma, Curcumae Radix* and *Curcumae Rhizoma* on blood rheology is shown in Table 8.

**TABLE 8:** Pharmacological parameters of effects of *Curcumae Longae Rhizoma*, *Curcumae Radix* and *Curcumae Rhizoma* on hemology.

Active extract	Animal models	Inducers	Experimental grouping, dosage, and administration method	Result	References
*Curcumae Longae Rhizoma* extract	New Zealand Rabbit and Diastolic Isolated Rat Thoracic Aorta	KCl	normal control group, sodium ferulate groups (15, 30, 60 mg/mL), different components of turmeric groups (6, 12, 24 mg/mL)	platelet aggregation and contraction of isolated rat thoracic aortic rings↓	Lei (2013)
*Curcumae Radix* ethanol extract	Large eared white rabbit, both male and female, 3–5 months old, weighing 2.0–2.5 kg	ADP	normal control group, *Curcumae Radix* ethanol extract 5 mL/kg, aspirin, 20 mg/kg, (ig)	platelet aggregation↓	Jiang et al. (2015)
*Curcumae Radix* extract	Rat carotid artery thrombosis model, SD rat, male, SPF grade, weight (200 ± 20) g	FeCl3	Sham surgery group, model group, positive control group, *Curcumae Radix* extract (50, 100, 150 mg/kg), (ig)	TXA2↓, PGI2↑, PT↑, APTT↑, platelet aggregation↓, TXB2↑、6-K-PGF1α↓	Su et al. (2019)
*Curcumae Rhizoma* extract	Tail thrombosis model in mice and vein thrombosis model in rats	Collagen - adrenaline, carrageenan	normal control group, Xuesaitong 60 mg/kg, *Curcumae Rhizoma* extract (1.5, 3.0, 6.0 g crude drug/kg), (ig)	Number of thrombotic black tailed animals↓, black tailed length↓, NO↑, 6-keto-PGF1α↑, ET-1↓, TXB2↓, Whole blood viscosity↓, plasma viscosity↓	Chen et al. (2018a), Chen et al. (2018b)
*Curcumae Rhizoma* extract	A rat model of ischemic stroke	suture-occluded method	Sham surgery group, model group, nimodipine 2 g/kg, *Curcumae Rhizoma* extract (4, 8, 16 g/kg), (ig)	Cerebral infarction volume percentage↓, brain water content↓, MDA↓, NO↓, SOD↑	Wu et al. (2013)

##### 2.4.1.2 Influence on blood glucose and blood lipid

It is estimated that 20%–25% of the global population have been diagnosed with metabolic syndrome (MetS); this is a chronic disease characterized by abnormalities in lipid metabolism, hypertension, hyperglycemia, and obesity. Furthermore, MetS can lead to abnormalities in the metabolic system, which can in turn induce the development of other diseases (Alberti et al., 2005). Lekshmi et al. (Lekshmi et al., 2012) evaluated the antidiabetic capacity of turmeric volatile oil by the α-glucosidase inhibition test and amylase inhibition test. Analysis showed that turmeric volatile oil increased the inhibitory ability of α-glucosidase (IC_50_ = 0.28 ±0.05μg/mL ) and α-amylase (IC_50_ = 24.5 ±4.8μg/mL ). Furthermore, the inhibition effect of glucosidase was more significant than that of the positive control drug (acarbose). In addition, research has shown that turmeric volatile oil has certain alleviating effects on hyperlipoidemia and reduces lipid-induced oxidative stress, platelet activation, and vascular dysfunction (Singh et al., 2013). In addition, turmeric volatile oil resulted in a significant decrease in the levels of TC, LDL-C, and TAG in the blood and livers of mice with high cholesterol (*p* < 0.001, *p* < 0.05). Furthermore, the levels of HDL-C were elevated (*p* < 0.05) and the levels of plasma MDA were decreased (*p* < 0.05), thus exerting lipid-lowering effects that were similar to the efficacy of the clinical drug ezetimibe. In addition, turmeric volatile oil significantly reduced the polymerization of ADP-, collagen-, and AA, and attenuated the phosphorylation of tyrosin in platelet proteins, thus resulting in antiplatelet aggregation efficacy, The anti-hyperlipidemic effect of volatile oil may be mediated through the regulation of PPARa, LXRa, and genes related to lipid metabolism and transport. Li et al. (Li et al., 2015) used a metabolomics approach based on nuclear magnetic resonance hydrogen spectroscopy (^1^H-NMR) and mass spectrometry (MS) to study the effect of curcumin on high-fat diet-induced hyperlipidemic mice. Analysis showed that curcumin achieved lipid-lowering effects via several metabolic pathways, including glycolysis and gluconeogenesis, creatine metabolism, ketone bodies and cholesterol synthesis.

**FIGURE 3:**
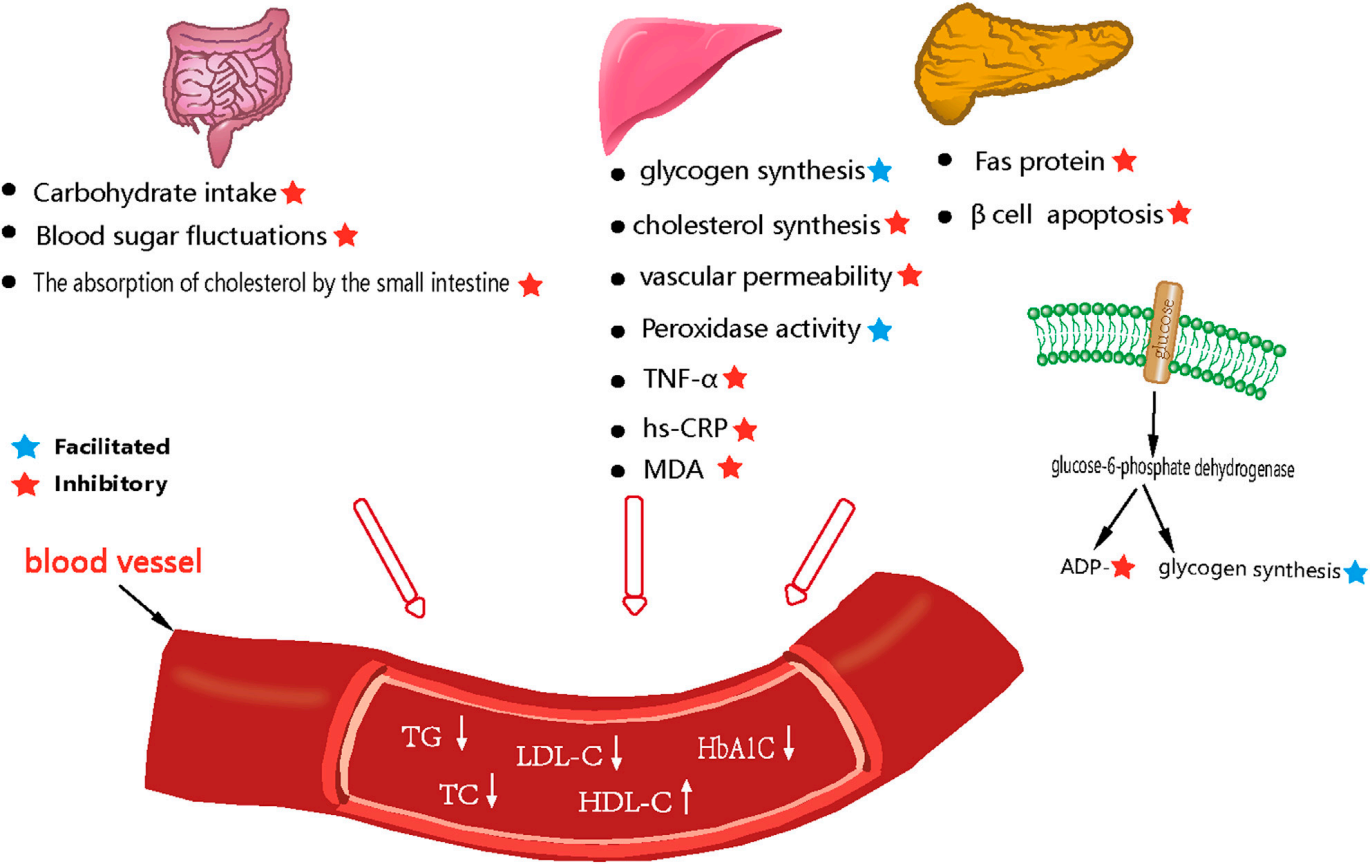
Mechanism diagram of reducing blood glucose and blood lipid levels in *Curcumae Longae Rhizoma, Curcumae Radix and Curcumae Rhizoma*.

Cai et al. (Cai et al., 2017) investigated the effects and mechanism of action of the sesquiterpenoid compound curcumolide from Wenyu-jin on a streptomycin-induced mouse model of diabetes. Diabetes was induced by an intraperitoneal (ip) injection of freshly prepared streptomycin (55 mg/kg). Analysis showed that curcumolide activated the NF-κB pathway and attenuated diabetic retinal vascular permeability and vascular white matter sludge, and reduced the overexpression of TNF-α and ICAM-1 in the diabetic retina. In another study, Xu et al. (Xu et al., 2009) investigated the antioxidant capacity of *Curcumae Rhizoma* polysaccharide and lovastatin. Experiments were divided into a blank control group, a lovastatin group (3.0 mg/kg), and *Curcumae Rhizoma* polysaccharide Ⅰ (300 mg/kg), II (600 mg/kg), Ⅲ (900 mg/kg) dose groups for 3 weeks. The blank control group was injected with the same volume of saline. Analysis showed that compared with the model control group, the levels of TC, TG, and LDL-c in the rats in the *Curcumae Rhizoma* polysaccharide-treated groups were reduced to close to normal level while the levels of HDL-c in the serum, and the activity of antioxidant enzymes, were significantly elevated. These results indicated that the protective effect of *Curcumae Rhizoma* polysaccharide on oxidative damage was comparable to that of lovastatin, and that this herb might play a protective role by regulating the degree of lipid peroxidation and by enhancing the antioxidant defense system. The pharmacological parameters describing the effects of *Curcumae Longae Rhizoma, Curcumae Radix* and *Curcumae Rhizoma* on blood glucose and blood lipids are shown in Table 9. In summary, it is highly evident that *Curcumae Longae Rhizoma, Curcumae Radix* and *Curcumae Rhizoma* can induce hypoglycemic and hypolipidemic effects by reducing the absorption of carbohydrates in the small intestine, vascular permeability, cholesterol synthesis, and apoptosis of β-cells; the associated mechanisms are shown in Figure 3.

**TABLE 9:** Pharmacological parameters of effects of *Curcumae Longae Rhizoma*, *Curcumae Radix* and *Curcumae Rhizoma* on blood glucose and blood lipid.

Active extract	Animal models and inducers	Experimental grouping, dosage and administration method	Result	References
turmeric volatile oil	-	① 10 μl contains turmeric volatile oil extract or positive drug acarbose (10–100 μg ), plus 50 μl ,50% 3,5-dinitrosalicylic acid ② Add α-glucosidase (20 mL, 1 U/mL) to 25–500 extract/acarbose and dilute to 500 μl with phosphate buffer solution	increase the inhibitory ability of α-glucosidase (IC_50_ = 0.28 ±0.05μg/mL ) and α-amylase (IC_50_ = 24.5 ±4.8μg/mL )	Lekshmi et al. (2012)
turmeric volatile oil	Hyperlipidemic mice (high cholesterol diet)	blank control group, model group, turmeric volatile oil (30, 100, 300 mg/kg), ezetimibe group 1 mg/kg/d, (po)	TC↓, LDL-C↓, TAG↓, HDL-C↑, MDA↓, ADP-↓, Collagen ↓, AA ↓, tyrosine phosphorylation ↓	Singh et al. (2013)
turmeric volatile oil	Rat hyperlipidemia model (high fat diet)	blank control group, Model group, Xuezhikang 115 mg/kg, turmeric volatile oil (100, 300 mg/kg), (ig)	TC↓, TG↓, LDL↓, Restore kidney and heart weight, liver fat deposition↓	Lee et al. (2018)
curcumin	Hyperlipidemic mice (high fat diet)	blank control group, Model group, lovastatin 30 mg/kg、curcumin group (40、80 mg/kg), (po)	TC↓, TG↓, LDL-c↓, HDL-c↑	Li et al. (2015)
NCB-02 (curcumin standardized formulations)	Patients with type 2 diabetes	NCB-02 group (contain curcumin 150 mg), atorvastatin 10 mg, (po)	MDA↓, ET-1↓, IL-6↓, TNF-α↓	Usharani et al. (2008)
curcumin	Patients with type 2 diabetes	Placebo group 500 mg carboxymethyl cellulose capsules, treatment group 500 mg Curcumin+5 mg piperine, (po)	TG↓, FVG↓, glycosylated hemoglobin (HbA1c)↓	Neta et al. (2021)
curcumenolactone	Diabetes model mice (streptomycin)	blank control group, model group, Model group + triamcinolone acetonide、curcumenolactone group (0, 2.5, 5, 10, 20 μM )	vascular leakage↓, white blood cell stasis↓, TNF-α↓, ICAM-1↓	Cai et al. (2017)
*Curcumae Rhizoma* oil	Atherosclerotic rats (high-fat diet)	experimental group injected 40 mg/kg *Curcumae Rhizoma* oil glucose injection into mice, blank control group (ip)	TC↓, TG↓, LDL-C↓, IL-2↓, hs-CRP↓, TNF-α↓, HDL-C↑	Liu et al. (2016)
*Curcumae Rhizoma* polysaccharide	High fat rats (high fat feed)	blank control group, lovastatin 3.0 mg/kg, *Curcumae Rhizoma* polysaccharide (300, 600, 900 mg/kg), (po)	TC↓, TG↓, LDL-c↓, HDL-c↑, antioxidant enzyme activity↑	Xu et al. (2009)
*Curcumae Rhizoma* polysaccharide	Type 2 diabetes rats (high-fat diet combined with ip injection of streptozotocin)	Model group, *Curcumae Rhizoma* polysaccharide (0.5, 1.0, 2.5 g/kg), blank control group (ip)	TC↓, TG↓, Pancreatic Fas protein↓, pancreatic islets β Cell apoptosis↓	Xiao et al. (2015)

#### 2.4.2 Effects on the nervous system

Neurodegenerative diseases, such as epilepsy, Alzheimer’s disease (AD), depression, Parkinson’s disease (PD) and others, are characterized by a complex pathogenesis and rapid onset; these diseases represent a significant challenge for both the patient and clinician. Eun et al. (Eun et al., 2017) investigated the effects of turmeric extract on memory dysfunction in two brain cell lines (C6 rat glioma cells and BV2 mouse microglial cells) and a scopolamine-induced mouse model of memory dysfunction. Turmeric extract inhibited the time taken for t-BHP and H_2_O_2_-induced cellular damage to occur while also reducing the production of pro-inflammatory mediators, including NO, TNF-α, PGE2, iNOS, and COX-2. Furthermore, turmeric extract attenuated scopolamine-induced memory impairment, inhibited the activity of AChE, and promoted the activation of CREB and the expression of brain-derived neurotrophic factor in experimental mice. Li et al. (Li H. et al., 2022) found that curcumin improved sevoflurane-induced neuronal cell injury mainly by inhibiting the inflammation, oxidative stress and neuronal apoptosis of the hippocampus. Yu et al. (Su et al., 2022) used the Morris water maze test and Western blotting to investigate the effects of curcumin on memory function and GSK3β protein expression in the hippocampus of AD rats. Analysis showed that the evasion latency time in the curcumin group was significantly increased, the number of crossing platforms was reduced (*p* < 0.05), and the expression of GSK3β protein in the hippocampus was reduced, thus significantly ameliorating neuronal cell damage in the rat model of AD. In another study, Fikry et al. (Fikry et al., 2022) investigated the potential protective influence of curcumin on the cerebellum of rats with rotenone‐induced PD. Analysis showed that curcumin attenuated neurotoxic effects and degenerative histological changes while alleviating induced oxidative stress in the cerebellar cortex of the rat model of PD. Therefore, curcumin may exhibit neuroprotective effects against the development of cerebellum‐related PD symptoms.

Wang et al. (Wang et al., 2016) used β-amyloid (Aβ25-35) to establish an Aβ25-35 SPF-grade mouse model of dementia and investigated the effects of *Curcumae Radix* extracts with different solvents. Analysis showed that aqueous, volatile oil and ethanol extracts of *Curcumae Radix* significantly improved the symptoms of AD mice. Qi et al. (Qi et al., 2017) investigated the effect of Wenyu-jin volatile oil on tau protein phosphorylation in a SPF mouse model of AD and also studied the mechanisms involved. Analysis showed that the phosphorylation level of tau protein (Thr231 and Ser404) in the hippocampus of the rats in the high-dose group of Wenyu-jin volatile oil was significantly reduced (*p* < 0.05), and that the levels of phosphorylation of PI3K and Akt were significantly increased (*p* < 0.05), thus demonstrating that the protective effects of the volatile oil of Wenyu-jin on AD mice might be related to the PI3K/Akt signaling pathway. Li et al. (Li et al., 2010) investigated the effect of an aqueous extract of *Curcumae Rhizoma* on monoamine neurotransmitters in different parts of the male rat brain. Analysis concluded that the aqueous extract of *Curcumae Rhizoma* promoted the secretion of catecholamine (CA), norepinephrine (NE), and 5-hydroxytryptophan (5-HT) in different parts of the brain tissue. Yu et al. (Yu et al., 2016) investigated the effect of an aqueous extract of *Curcumae Rhizoma* on the expression of neural cell adhesion molecule (NCAM), sialyltransferase ST8SiaII, ST8SiaIV, Fyn, and focal adhesion kinase (FAK) mRNA in the brains of offspring derived from rats with blood stasis or normal pregnancy when compared with a normal control group. The expression levels of ST8SiaII, p-FAK, NCAM, and p-Fyn protein in the hippocampus were significantly downregulated (all *p* < 0.05); this may represent one of the mechanisms underlying neurodevelopmental toxicity. Information on the parameters reported by pharmacological studies on the effects of *Curcumae Longae Rhizoma, Curcumae Radix* and *Curcumae Rhizoma* on the nervous system is shown in Table 10.

**TABLE 10:** Pharmacological parameters of effects of *Curcumae Longae Rhizoma*, *Curcumae Radix* and *Curcumae Rhizoma* on nervous system.

Active extract	Experimental type	Animal models	Inducers	Experimental grouping, dosage and administration method	Result	References
*Curcumae Longae Rhizoma* extract	*In vivo* and *In vitro*	Two types of brain cell lines (C6 rat glioma cells and BV2 mouse microglia) and memory impairment model mice	scopolamine	blank control group, model group, donepezil 5 mg/kg, *Curcumae Longae Rhizoma* extract (50, 100, 200 mg/kg), (po)	Cell damage time↓, pro-inflammatory mediators↓, including NO↓, TNF-α↓, PGE2↓, iNOS↓, COX-2↓, AChE↓, CREB↑, brain-derived neurotrophin↑	Eun et al. (2017)
curcumin	*In vivo*	SPF level cognitive impairment model rats	sevoflurane	Blank control group, model group, Curcumin group (200, 300 mg/kg), (ig)	IL-6↓, IL-1β↓, TNF-α↓, MDA↓, SOD↑, GSH-Px↑, Bax↓, Bcl-2↑	Li et al. (2022b)
curcumin	*In vivo*	AD model, 9-month-old male SPF grade C57BL transgenic mice, weighing 20–30 g	-	model group, curcumin group (200 mg/kg, the concentration is 25 mg/mL), (ig)	number of times crossing platforms↑, PICALM↓, GAP-43↑	Su et al. (2022)
curcumin	*In vivo*	AD model mice	Aβ_1-42_	Sham surgery group, model group, curcumin group (150, 300 mg/kg), (ig)	escape latency ↓, number of times crossing the platform↑, GSK3β↓	Yu et al. (2022)
curcumin	*In vivo*	Spontaneous intracerebral hemorrhage model rats	Bacterial collagenase	negative control, curcumin group (120, 480 mg/kg)	caspase-3mRNA↓, Relative ratio of Bcl-2 to Bax↑	Xu et al. (2021)
curcumin	*In vivo*	PD model in mice	rotenone	blank control group, model group (rotenone 3 mg/kg/day), model + curcumin group (30 mg/kg), curcumin group (30 mg/kg), (ip)	The active of AChE↑, MDA↓, GSH↑, SOD↑	Fikry et al. (2022)
*Curcumae Radix* extract	*In vivo*	Mouse dementia model	Aβ_25-35_	Sham surgery group, model group, Shenzhiling oral liquid group (2.6 mL/kg), donepezil group (1.3 mg/kg), and different solvent turmeric extract groups (water extract 0.16, 0.48 g/kg, ethanol extract 0.04, 0.08, 0.24 g/kg, petroleum ether extract 0.11, 0.33 g/kg, N-butanol extract 0.06, 0.18 g/kg, ethyl acetate extract 0.06、0.18 g/kg, volatile oil extract 0.016、 0.048 g/kg), (ig)	Spontaneous alternating reaction rate↑, swimming time↓, total swimming distance↓	Wang et al. (2016)
*Curcumae Radix* extract	*In vivo*	Mouse behavioral despair model (clean grade ICR mice, 18–22 g)	-	Fluoxetine hydrochloride group (26 mg/kg), Alcohol extract group (330 mg/kg), Petroleum ether extract group (70 mg/kg), Ethyl acetate extract group (40 mg/kg), N-butanol extract group (110 mg/kg), Water extract group (100 mg/kg), Blank control group (ig)	Forced tail suspension time↓, forced swimming time↓	Zhao et al. (2011)
*Curcumae Radix* volatile oil	*In vivo*	Alzheimer’s disease (AD) model mice	Aβ_25-35_	Sham surgery group, model group, Shenzhiling group (2.6 mL/kg), and Curcumae Radix volatile oil group (6.0, 18.0 mg/kg), (ig)	Tau proteins (Thr231 and Ser404) phosphorylation↓, PI3K↑, Akt phosphorylation↑	Qi et al. (2017)
*Curcumae Rhizoma* extract	*In vivo*	Rats (Wistar rats, male, weight 190–210 g)	-	*Curcumae Rhizoma* extract group (0.81, 2.43, 7.29 g crude drug/kg), Blank control group, (ig)	CA↑, NE↑, 5-HT↑	Li et al. (2010)
*Curcumae Rhizoma* water extract	*In vivo*	Blood stasis syndrome and normal pregnant rats (Wistar rats)	Compound method of adrenaline hydrochloride and ice water bath	Blank control group、model group, *Curcumae Rhizoma* water extract (1.4, 2.8, 5.6 g/kg), (ig)	ST8SiaII↓, p-FAK↓, NCAM↓, p-Fyn↓	Yu et al. (2016)
Curdione	*In vivo*	A mouse model of cerebral ischemia-reperfusion injury (SPF grade 2-month-old C57BL/c mice weighing 18–22 g)	suture-occluded method	Sham surgery group, model group, low molecular weight heparin sodium group 1 mg/kg, Curdione group (20 mg/kg, 60 mg/kg), (ig)	Escape latency↓, number of platform crossings↑, behavioral score↓, cerebral infarction rate↓, brain tissue water content↓, 6-keto-PGF1α↑, 6-keto-PGF1α/TXB2 ratio↑, cAMP↑, p-CREB↑	Li et al. (2020)

#### 2.4.3 Effects on respiratory system

Respiratory diseases mainly include asthma, chronic obstructive pulmonary disease, and pneumonia. Most of the current treatments involve leukotriene inhibitors, glucocorticoids and bronchodilators, although these methods do not solve the main causative problem. Thus, the discovery of safe and effective therapeutic drugs has become a major challenge (Glass and Rosenthal, 2018; Liu Y. et al., 2023). Manarin et al. (Manarin et al., 2019) investigated the effects of turmeric extract on asthma; patients received either turmeric extract or placebo (approximately 30 mg/kg/d). After 6 months of treatment, analysis showed that the treatment group taking turmeric extract had a lower frequency of nocturnal awakenings and a lower frequency of short-acting β-adrenergic agonists when compared to the placebo group of asthma patients; thus, turmeric extract was effective in controlling asthma exacerbations. In addition, Houssen et al. (Houssen et al., 2010) evaluated the efficacy of *Boswellia serrata*, *Glycyrrhiza glabra*, and *Curcumae Longae Rhizoma* as a combination of natural leukotriene inhibitors, anti-inflammatory agents, and antioxidants, respectively, for the control of bronchial asthma by randomizing patients with bronchial asthma into a treatment group (150 mg boswellic acid, 50 mg licorice extract, and 15 mg curcumin) and placebo groups. After 4 weeks, 5 mL of blood were collected from each patient, and lung function was assessed by measuring the levels of leukotrienes, NO, and MDA in the plasma of all the patients participating in the stud. Compared to the placebo group, patients of the treatment group showed a significant reduction in the levels of leukotrienes, NO, and MDA in plasma; thus, Boswellia serrata, glycyrrza glabra, and *Curcumae Longae Rhizoma* extracts had significant therapeutic effects on bronchial asthma. In another study, Sun et al. (Sun, 2014) investigated the effects and mechanisms of curcumin on lung injury induced by one-lung ventilation in rabbits. Twenty-four New Zealand white rabbits were randomly divided into a two-lung ventilation group (TLV group), a one-lung ventilation group (OLV group), and a curcumin group (40 mg/kg). The TLV group and the OLV group were given 2 mL of 1% carboxymethylcellulosesodium and the administration was begun with the corresponding dose 7 days prior to the experiment, and was administered twice every day. Analysis showed that the oxygenation index at T3 was elevated in the curcumin group when compared with the OLV group (*p* < 0.05), the wet/dry weight ratio of the right and left lungs was reduced in the curcumin group (*p* < 0.05), SOD activity was elevated in the lung tissues of the curcumin intervention group (*p* < 0.05), and the levels of MDA were reduced (*p* < 0.05). Moreover, the levels of Nrf2 protein in the lung tissue of the curcumin group were elevated (*p* < 0.05); thus, curcumin exerted a protective effect on single-lung ventilation-induced lung injury in rabbits by regulating the Nrf2/ARE signaling pathway. Information on the parameters reported by pharmacological studies on the effects of *Curcumae Longae Rhizoma*, *Curcumae Radix* and *Curcumae Rhizoma* on the respiratory system is shown in Table 11.

**TABLE 11:** Pharmacological parameters of effects of *Curcumae Longae Rhizoma*, *Curcumae Radix* and *Curcumae Rhizoma* on respiratory system.

Active extract	Animal models	Dosage	Result	References
turmeric extract	Asthmatic patients	placebo group and turmeric extract group (500 mg/d for patients aged 7–10, 750 mg/d for patients aged 11–14, and 1,000 mg/d for patients aged 15–18)	Night awakening frequency↓, short effect β- Frequency of use of adrenergic receptor agonists↓	Manarin et al. (2019)
Boswelliaserrata, glycyrrzaglabra and *Curcumae Longae Rhizoma*	Patients with bronchial asthma	treatment group (containing 150 mg of mastic acid, 50 mg of licorice extract, 15 mg curcumin) and placebo group	leukotriene↓、NO↓、MDA↓	Houssen et al. (2010)
curcumin	A rabbit lung injury model induced by single lung ventilation	double lung ventilation group, single lung ventilation group, Curcumin intervention group (40 mg/kg)	Oxygenation index↑, right and left lung wet/dry weight ratio↓、SOD↑、MDA↓、Nrf2↑	Sun (2014)

#### 2.4.4 Effect on digestive system

The digestive system, as one of the eight major systems in the human body, consists of two parts: the digestive tract and the digestive glands. The basic physiological functions of this system are the ingestion, transit and digestion of food, the absorption of nutrients, and the excretion of wastes. Digestive diseases include gastrointestinal disorders and disorders of the liver, gallbladder, spleen, and pancreas (Li et al., 2023). Li (Li, 2020) used a high-fat diet to establish an animal model of gallbladder cholesterol stones in C57BL/6 mice and then investigated the role and mechanism of curcumin in the prevention of cholecystolithiasis. Analysis showed that curcumin acted by inhibiting the hydrolysis of SREBP-2 precursor protein to modulate the expression of NPC1L1 in cells of the small intestine. Hanai et al. (Hanai et al., 2006) evaluated the therapeutic effect of curcumin on ulcerative colitis (UC) in clinical trials and found that curcumin combined with mesalamine had significantly better clinical efficacy than placebo + mesalamine in preventing recurrence, significantly relieving the clinical symptoms of UC and reducing the recurrence rate. Sugimoto et al. (Sugimoto et al., 2002) investigated the effect and mechanism of curcumin on a trinitrobenzene sulfonic acid-induced mouse model of colitis; 0.5%, 2.0%, and 5.0% curcumin was added to the feed of mice in the curcumin intervention group. Then, the levels of NF-κB in the colonic mucosa were detected by immunohistochemistry while RT-PCR was used to detect the expression of cytokine mRNA in colon tissues. When compared to the model group, curcumin reduced mortality in mice with colitis, inhibited IκB, induced the nuclear translocation of NF-κB into the epithelial nucleus, and reduced the infiltration of CD4^+^ T-cells. Thus, the authors concluded that curcumin could exert significant clinical therapeutic effects on mice with colitis by inhibiting the expression of pro-inflammatory cytokine mRNA and NF-κB activation in the colonic mucosa. Information on the parameters reported by pharmacological studies on the effects of *Curcumae Longae Rhizoma, Curcumae Radix* and *Curcumae Rhizoma* on the digestive system is shown in Table 12.

**TABLE 12:** Pharmacological parameters of effects of *Curcumae Longae Rhizoma*, *Curcumae Radix* and *Curcumae Rhizoma* on digestive system.

Active extract	Animal models	Inducers	Experimental grouping, dosage and administration method	Result	References
curcumin	Mouse gallbladder cholesterol gallstone model (C57BL/6 mice, 8 weeks old, around 20 g)	high-fat diet	Regular diet + CMC (sodium carboxymethyl cellulose), high-fat diet + CMC, high-fat diet + curcumin (200, 500, 1000 mg/kg/d), high-fat diet + piperine (20 mg/kg/d), high-fat diet + curcumin (500 mg/kg/d)+piperine (20 mg/kg/d), (ig)	Gallstone formation rate↓, gallbladder volume↓, cholesterol level↓、NPC1L1mRNA↓、SREBP-2mRNA↓	Li (2020)
curcumin	Patients with ulcerative colitis (UC)	-	In the control and treatment groups, patients were given mesalazine (1.5–3.0 g/d), median 2.25 g+2 g curcumin, (po)	Significant improvement in clinical activity index (CAI) and endoscopic index (EI)	Hanai et al. (2006)
curcumin	Mouse colitis model (7–8 week old male C57BL/6 and BALB/c mice, 21–23 g)	Trinitrobenzene sulfonic acid (TNBS)	Blank control group, model group, treatment group with different concentrations of curcumin (0.5%, 2.0%, 5.0% in feed), (po)	Mouse colitis mortality rate↓、NF-κB↓、CD4+T cell infiltration ↓, IL-6↓, TNF-α↓, IL-12↓, IFN-γ↓	Sugimoto et al. (2002)

### 2.5 Other conditions

Curcumin has been shown to have a therapeutic effect on damage incurred by several organs, including the liver, kidney, skin, hands, feet, and mouth (Hu et al., 2018), and has also been shown to have antiprotozoal, antispasmodic, and antidepressant effects (Araújo and Leon, 2001; Wang Y. et al., 2022). Gong et al. (Gong et al., 2022) found that curcumin protected retinal Müller cells from damage caused by ultraviolet light, mainly through the activation of the Nrf2 signaling pathway in Müller cells; this attenuated damage in a mouse model. Some researchers have proposed that curcumin can significantly elevate weight, reduce viral load and plasma D-dimer (D-D), the levels of neuron-specific enolase (NSE) and neuropeptide Y (NPY) in rats with severe hand-foot-and-mouth disease (Ming et al., 2022). Li et al. (Li et al., 2018) investigated the effects and mechanisms of ar-turmerone in a mouse model of imiquimod-induced psoriasis and concluded that ar-turmerone inhibited CD8^+^ T cell metastasis in the epidermis, decreased the expression of NF-κB, COX-2 and IL-6 levels, reduced the phosphorylation of p38MAPK, and downregulated the expression of IL-17, IL-22, and IL-23 mRNA.

The extracts of Wenyu-jin were shown to have a terminating effect on all periods of mouse pregnancy, although oral administration was ineffective. In addition, Wenyu-jin extract had a significant excitatory effect on the isolated uterus of non-pregnant or mice in the early stages of pregnancy; as the dose of Wenyu-jin extract increased, the stronger the excitatory effect on the uterus became (Zhang et al., 1983). In another study, Xie et al. (Xie et al., 2020) used biochemical and histopathological methods to evaluate the anti-liver fibrosis effect of Wenyu-jin extract and showed that Wenyu-jin extract inhibited the activation and proliferation of hepatic stellate cells and induced apoptosis by blocking the TGF-β/Smad signaling pathway and significantly up-regulating the level of MMP-2/TIMP-1. These data indicated that Wenyu-jin extract was involved in the degradation of the extracellular matrix, and also maintained the formation and production of the extracellular matrix. Liu et al. (Liu and Cheng, 2012) confirmed that the traditional Chinese medicine *Curcumae Rhizoma* can delay renal interstitial fibrosis in rats with unilateral ureteral obstruction, reduce the content of N-acetyl-β-aminoglucosidase and urinary protein in urine; its effect on the kidneys were comparable to that of Losartan. Liu et al. (Liu et al., 2018) investigated the therapeutic effect and mechanism of *C. kwangsiensis* extracts on psoriasis and showed that *C. kwangsiensis* extract reduced the dendritic cell expression of lymphatic homing chemokine receptor CCR7 and its ligand CCL21 while also significantly reducing the activity of pro-inflammatory cytokines (IL-12, IL-6 and IL-1β), as well as the proliferation of T-cells and the differentiation of Th1 and Th17 cells. Collectively, this data suggested that *C. kwangsiensis* extract could represent a potential drug for psoriasis.

### 2.6 Discussion

*Curcumae Longae Rhizoma*, *Curcumae Radix* and *Curcumae Rhizoma* all belong to the *Curcuma* species. Their chemical compositions are similar, although each has its own characteristics due to differences in medicinal components, geographic factors, and other factors. For example, *Curcumae Longae Rhizoma* is native to subtropical regions, and its curcuminoid content is much higher than that of *Curcumae Radix* and *Curcumae Rhizoma*; *C. kwangsiensis* contains the least amount of curcumin. Research has shown that the curcumin content of *Curcumae Longae Rhizoma* is higher than that of *Curcumae Radix* (Wang, 2014). Generally, rhizomes of the same species contain more curcuminoids and volatile oil compounds than their tubers.

In terms of clinical application, *Curcumae Longa Rhizoma* and *Curcumae Radix* are mainly used in raw products, the vinegar products of *Curcumae Rhizoma* can be used to enhance the role in removing blood stssis and eliminating mass. In terms of efficacy, *Curcumae Longae Rhizoma* is efficacious in promoting menstruation to relieve pain and is mostly used in the treatment of rheumatic diseases. *Curcumae Rhizoma* is efficacious at removing accumulation and alleviating pain, activating qi and blood circulation, and treating dyspepsia distending pain. *Curcumae Radix* is efficacious at activating qi and relieving depression, and activating blood circulation and alleviating pain (Qin et al., 2022b). In terms of medicinal properties, all three herbs can activating blood circulation and removing blood stasis, activating qi and alleviating pain, and can be used to treat blood stagnation, amenorrhea with abdominal mass. However, *Curcumae Longae Rhizoma* and *Curcumae Rhizoma* are both efficacious for the cold accumulation causing qi stagnation and blood stasis while *Curcumae Radix* is suitable for the treatment of depression stagnation due to blood heat. Modern pharmacological studies have shown that *Curcumae Longae Rhizoma* exerts mainly anti-inflammatory, analgesic, antioxidant, hypoglycemic, hypolipidemic, and antitumor effects while also improving blood rheology, antibacterial properties, protecting the nervous system, and other pharmacological effects (Wang and Xu, 2018). These properties can treat rheumatoid arthritis, cardiovascular and cerebrovascular diseases, neurodegenerative diseases, diabetes mellitus and other diseases with significant therapeutic effects. *Curcumae Radix* can regulate immune function, exert anti-inflammatory effects, protect the nervous system, protect the liver and promote gallbladder function, thus improving blood rheology and exerting anti-tumor effects. This herb is mainly used in the treatment of hepatobiliary system diseases, chronic gastritis, hepatitis, psoriasis, cardiovascular and cerebrovascular diseases, tissue contusion, lithiasis disease, and cyclomastopathy. *Curcumae Rhizoma* is mainly used for the clinical treatment of various malignant tumors and has significant therapeutic effects on the uterus. When combined with *Carthami flos*, *Persicae semen* and other herbs, it also has significant curative effects on endometriosis, dysmenorrhea, chronic pelvic inflammatory disease and other gynecological diseases. In contrast, *Curcumae Rhizoma* is also efficacious in removing stagnation; clinical preparations containing *Curcumae Rhizoma* and “xiao’er Huashi Koufuye” are mainly used for the treatment of nervosa, dyspepsia, and constipation in children (Tong et al., 2020).

*Curcuma* species exert significant anti-inflammatory and antioxidant effects mainly by modulating oxidative stress as well as NF-κB and other pathways, thus affecting the levels of factors such as TNF-α, IL-6, IL-1β, IL-8, NO, and COX-2 *in vivo* to exert anti-inflammatory effects. These herbs exert antioxidant effects by regulating the levels of SOD, GSH, MDA, and ROS (Ak and Gülçin, 2008). *Curcumae Longae Rhizoma*, *Curcumae Radix* and *Curcumae Rhizoma* all exhibit some effects on the blood system; however, *Curcumae Rhizoma* has a stronger blood-activating effect than turmeric. Research has been shown that there was no significant difference between the curcuminoid compounds in turmeric and *Curcumae Rhizoma* in terms of antiplatelet aggregation and vascular dilatation (Lei, 2013). However, the differences produced by their volatile oil components is very obvious; the inhibitory effects of turmeric volatile oil components on platelet aggregation is weaker, thus showing that the volatile components are the potential material basis for the efficacy of blood-activation. *Curcumae Radix* mainly exerts its efficacy of purging heart and cooling blood by regulating lipid metabolism, plasma lipoprotein levels, platelet activation, oxidative stress reactions, apoptosis and other processes. Curcuminoids and volatile oil compounds (e.g., curcumin, curcumol, curdione, β-elemene, curzerenone, and germacrone) in *Curcuma* species have certain anti-tumor effects, of these, β-elemene is considered to be a broad-spectrum antitumor agent and is known to play a therapeutic role in the treatment of a variety of malignant tumors and has few side effects in humans (Chen et al., 2021). Overall, *Curcumae Longae Rhizoma* is mostly known for its anti-inflammatory and antioxidant effects while *Curcumae Radix* is mainly known for its effects on the cardiovascular system; *Curcumae Rhizoma* is mainly known for its anti-tumor effects.

There is no systematic exposition of the herbs of the *Curcuma* species. Most of the existing research focuses on investigating single pharmacological effects of traditional Chinese medicine or the pharmacological effect of an active ingredient within such medicine (Yuan et al., 2015). Future research should focus on other active ingredients to comprehensively evaluate the pharmacological effects of medicinal materials. In terms of the quality control of medicinal materials, the Chinese Pharmacopoeia only stipulates the content of volatile oil and curcumin of *Curcumae Longae Rhizoma*, and the content of the volatile oil of *Curcumae Rhizoma*, but does not stipulate the content of *Curcumae Radix*. In order to more comprehensively evaluate the quality standards of medicinal materials, it is important to include a wider range of scientific and technical methods.
